# The Influence of pH on Long-Range Electron Transfer and Proton-Coupled Electron Transfer in Ruthenium-Modified Azurin

**DOI:** 10.3390/molecules30030472

**Published:** 2025-01-22

**Authors:** Nikta Ghazi, Jeffrey J. Warren

**Affiliations:** Department of Chemistry, Simon Fraser University, 8888 University Drive, Burnaby, BC V5A 1S6, Canada

**Keywords:** electron transfer, proton-coupled electron transfer, reorganization energy, semi-classical theory, copper proteins, azurin

## Abstract

Long-range electron transfer (ET) is an essential component of all biological systems. Reactions of metalloproteins are important in this context. Recent work on protein “charge ladders” has revealed how the redox state of embedded metal ions can influence the ionization of amino acid residues at protein surface sites. Inspired by these observations, we carried out a variable pH investigation of intramolecular ET reactions in a ruthenium-modified protein system built on azurin from *Pseudomonas aeruginosa*. We also generate a Pourbaix diagram that describes the variable pH redox behavior of a Ru model complex, Ru(2,2′-bipyridyl)_2_(imidazole)_2_(PF_6_)_2_. The intramolecular ET rate constants for the oxidation of azurin-Cu^+^ by flash-quench-generated Ru^3+^ oxidants do not follow the predictions of the semi-classical ET rate expression with fixed values of reorganization energy (λ) and electronic coupling (H_DA_). Based on the pH dependence of the Ru^3+/2+^ redox couple, we propose a model where pure ET is operative at acidic pH values (≤ 7) and the mechanism changes to proton-coupled electron transfer at pH ≥ 7.5. The implications of this mechanistic proposal are discussed in the context of biological redox reactions and with respect to other examples of intramolecular ET reactions in the literature.

## 1. Introduction

The workings of biological redox systems involving electron flow through and between proteins presents an intricate puzzle to scientists [1,2,3,4,5,6]. Biological electron transfer (ET) processes have a large number of variables that can influence the function and the malfunction of reactions that are crucial for life, as discussed in the above (and many other) references. One key variable is protonation, or proton transfer (PT), which is another of the most fundamental chemical reactions. Such reactions where ET and PT occur together (simultaneously or sequentially) have been termed “proton-coupled electron transfer” (PCET) [7,8,9,10,11]. In the end, an ongoing challenge to understanding biological redox chemistry is to understand how protons influence the rates, energetics, and selectivity of redox reactions.

Recent research has highlighted the importance of the local environment of cofactors in modulating the properties of redox cofactors and their ET reaction. One earlier example is the hydrogen-bonded tyrosine_Z_ in photosystem II, which facilitates reversible PCET in the net water oxidation reaction [12,13,14]. In work on *Pseudomonas aeruginosa* azurin, the proximity of charged amino acid residues near a redox-active tyrosine also influences its thermodynamic properties [15,16]. Such observations also have been made in peptide-based models for the study of redox-active amino acid residues [17,18,19,20,21]. With respect to the embedded azurin-Cu ion, second sphere (i.e., non-coordinated) amino acid residues also can exert influence on the redox properties of the Cu site [22]. In general, the above examples can be classified as short-range effects, but comparatively less is known about the long-range influence of protein structures and amino acid composition on embedded sites. In this work, we set out to address some of these questions, in particular with respect to pH and ionization of surface sites.

The identity and composition of protein surface sites are known to influence the properties of embedded cofactors. For example, a series of azurin variants show how hydrophobic amino acid residues can systematically affect Cu^2+/1+^ reduction potentials [23]. While it may not be surprising that amino acid variations influence embedded cofactors (even at a distance), there are numerous examples of pH-dependent reduction potentials, especially in metalloproteins. A few examples include azurin [24], ferredoxins [25], Rieske proteins [26], b-type cytochromes [27,28], and c-type cytochromes [29,30,31]. That list is far from comprehensive, and one can reasonably assume that nearly all redox proteins will have redox properties that depend on pH. In azurin, for example, it has been proposed that the ionization state of (mainly) surface amino acid residues influences the properties of the cofactor [24,32].

Recent work from Shaw and co-workers demonstrated that a change in redox state of an embedded cofactor influences the ionization state of surface amino acid residues [33,34,35]. Such behaviors are called “charge regulation”, wherein the unit change in charge of the embedded cofactor is compensated by changes in the p*K*_a_ of other amino acid residues [36,37,38,39]. In this way, most redox proteins are better described as PCET proteins. Stated more explicitly, redox cycling of metal cofactors can involve proton transfer reactions at protein surfaces, including sites that are distant from the metal ion. In addition to thermodynamics considerations, PCET reactions can have different associated reorganization energy (cf. examples from theoretical treatments of PCET in ref. [40]). In short, the coupling of ET reactions and proton transfer reactions can have a wide range of effects on the rate(s) of biological redox chemistry.

**Figure 1:**
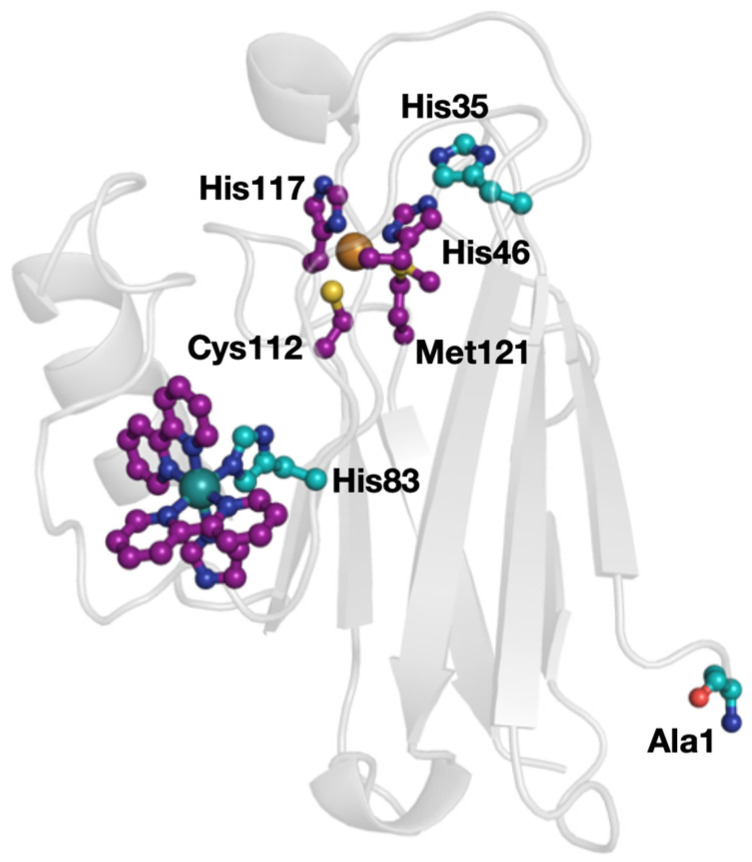
X-ray structure of Ru(2,2′-bipyridyl)_2_(imidazole)-modified azurin (PDB ID: 1BEX). The primary ligands at the Cu ion are shown (His46, Cys112, His117, Met121) and the Ru complex is placed at His83. The amino acid residues implicated in charge regulation are shown in teal and other key sites are in purple.

Azurin is one of the most studied models of biological ET reactions. As noted above, its pH-dependent redox chemistry has been described [24], its charge regulation theoretically treated [33], as well as experimentally probed [32]. Residues His35 and His83 were identified as sites that change their p*K*_a_ upon a redox change in the Cu ion. That work is in good agreement with computation work from Ullmann and co-corkers, who explored the protonation states of the two histidine sites [32]. While the discrete order of redox and protonation reactions was not determined, the two sites are tightly coupled. Herein, we use the known Ru-His83-modified azurin variant (Figure 1) to explore the pH dependence of intramolecular ET. We employ the well-known [41] Ru(bpy)_2_(imidazole)^3+/2+^ system to initiate intramolecular ET reactions (bpy = 2,2′-bipyridyl).

## 2. Results

The pH-dependent properties of azurin and of the model complex Ru(bpy)_2_(im)_2_^2+^ were first investigated. The azurin Cu^2+/1+^ redox couple has a known pH dependence, but in a different buffer system [24]. Although the buffer system is likely to be only a minor contribution, we also collected data using an APB (acetate–phosphate–borate) buffer to ensure that a different buffer did not influence the potentials. While we did not undertake studies at the same level of detail as that of the past report, the measured potentials at pH values between 4 and 8 were in accord with that of a previous work [24]. The potentials for the other pH values indicated below are calculated using the model set out in reference [24]. Additional details are set out in the Supporting Information.

**Figure 2:**
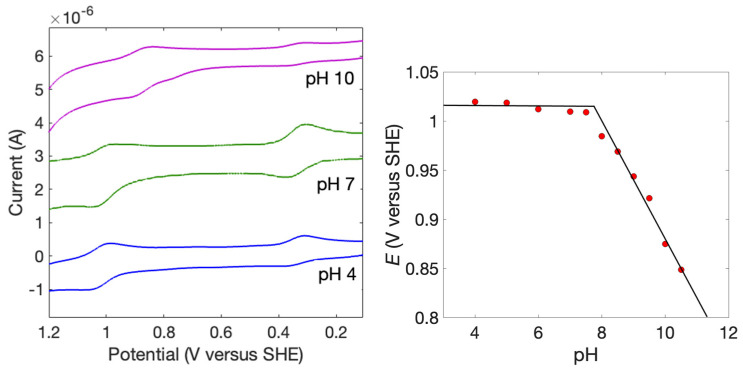
**Left**: Representative cyclic voltammograms of [Ru(bpy)_2_(im)_2_](PF_6_)_2_ in APB buffer at the pH values indicated. The wave at 0.3 V is for the [Co(bpy)_3_]^3+/2+^ internal standard. **Right**: Pourbaix diagram for [Ru(bpy)_2_(im)_2_](PF_6_)_2_ in APB buffer.

Next, the pH-dependent electrochemistry of the Ru(bpy)_2_(im)_2_^3+/2+^ couple was investigated using cyclic voltammetry (Figure 2). Cobalt(II)tris(2,2′-bipyridyl)(PF_6_)_2_ was used as a pH independent internal standard (*E* = 0.30 V versus SHE [42]). The pH-dependent photophysical properties of the Ru^2+^ complexes have been described at pH values between 10 and 14 [43], but not the pH dependence at lower pH values. To the best of our knowledge, the pH-dependent reduction potentials are not reported, though the Ru^3+/2+^ potential at pH 3 is known [44]. That value (1.00 V versus SHE) is in agreement with our measured value between pH 4 and 7 (*E* = 1.014 ± 0.005 V). At pH values higher than 7.5, the Ru^3+/2+^ decreases in potential by 55 mV per decade, which is close to the value expected for a 1H^+^/1e^−^ process (59 mV per pH). From these data, the p*K*_a_ of the Ru^3+^ complex is 7.6 ± 0.1, and the reported p*K*_a_ for the corresponding Ru^2+^ complex is 11.9 ± 0.2 [43].

The modification and purification of azurin at His83 with a [Ru(bpy)_2_(im)]^2+^ label was carried out according to the literature using Ru(bpy)_2_CO_3_ for initial labeling and incubation in imidazole-containing buffer to fill the remaining coordination site [41]. The efficiency is about 80%, following purification. Successful modification was evinced by characteristic UV-Vis absorption peaks at 628, 490, and 350 nm, which are characteristic of the azurin Cu site and for the Ru complex. The spectrum of the Ru-labeled protein is shown in the Supporting Information (Figure S2). Intramolecular ET rates were determined using a home-built transient absorption spectrometer [45,46]. To induce excitation, the samples were exposed using a wavelength of 480 nm. This laser light was generated via an optical parametric oscillator (OPO) laser pumped with frequency-tripled (355 nm) light from a neodymium-doped yttrium aluminum garnet (Nd-YAG) laser. This experimental arrangement involved the utilization of a 10 mM concentration of [Ru(NH_3_)_6_]^3+^ as a quencher for reversible redox reactions.

**Figure 3:**
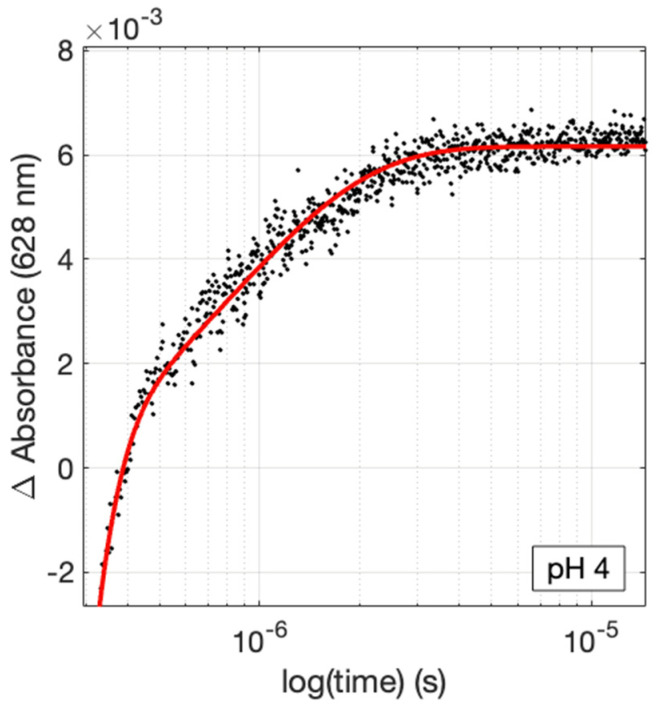
Example of time-resolved kinetics traces (628 nm) of Ru-His83 azurins. The concentrations for each trace were 30 µM protein in 20 mM APB buffer at the pH values indicated. The concentration of the quencher, Ru(NH_3_)_6_Cl_3_ was 12 mM. The apparent bleach at early times arises from luminescence from unquenched (residual) *Ru^II^. The pH values are as indicated in each panel.

Samples with varying pH values were probed using time-resolved transient absorbance spectroscopy to investigate the influence of pH on intramolecular ET kinetics. A typical flash-quench transient experimental set-up was used (see Supporting Information, Figure S3) with Ru(NH_3_)_6_Cl_3_ as a reversible ET quencher [47,48]. The kinetic traces were analyzed as described previously [15,16]. A representative kinetics trace in shown in Figure 3 and all kinetics traces are shown in the Supporting Information (Figure S4). The obtained ET rate constants are set out in Table 1. The driving forces (−∆G°) for the reactions at different pH values were computed based on the electrochemical data from above, and these values also are shown in Table 1.

## 3. Discussion

The starting point for rationalizing long-range intramolecular ET reactions is the semiclassical rate expression (Equation (1)). Here, ∆G° is the reaction driving force (given by the difference in formal reduction potentials), λ is the reorganization energy, *H_AB_* is the electronic coupling matrix element, and the other physical constants have their usual meanings. The value of λ for azurin is 0.7 to 0.8 eV [47,49,50,51], and here we use 0.7 eV, but it should be noted that our conclusions also hold if 0.8 eV is used. For *H_AB_*, the value at close contact (3 Å) is 186 cm^−1^, and the value at large distances (r) is determined using a square barrier model: *H_AB_* = (186 cm^−1^)·*exp* (0.5 βr). The value of the distance decay constant β is an empirical value for proteins (1.1 Å^−1^) [48,52].

(1) $$k_{ET}=\sqrt{\frac{4\pi^{3}}{h^{2}\lambda RT}}{H_{AB}}^{2}exp\left(-\frac{{\left(\Delta G{}^{\circ}+\lambda \right)}^{2}}{4\lambda RT}\right)$$

Using the above values as constants, and the experimentally determined driving forces (−∆G°), it is straightforward to compute rate constants for ET (*k*_ET,calc_) in RuHis83 azurin at each pH value (Table 1). We also provide corresponding values with a different value of λ in Table S2 (see Supporting Information). The agreement between experiment and theory is good at pH values between 4 and 8, but less so at pH 9 and 9.5. The calculated rate constants show that, in principle, ET should be fastest at pH 7, but this is not possible to confirm within experimental uncertainty. At pH values of 9 and above, the rate constants are predicted to be smaller in magnitude, but the discrepancy between experiment and theory is larger than that for the other pH values. Unfortunately, the stability of RuHis83-azurin at pH values about 9.5 is decreased, especially during irradiation in time-resolved spectroscopy experiments.

The above ET analysis (i.e., values in Table 1) does not account for all aspects of the pH dependence of the azurin protein and the Ru label; only the driving force (−∆G°) is include in the calculations of *k*_ET_. The azurin Cu^2+/+^ potential is pH-dependent between about pH 5.5 and 8, and the Ru potential is pH-dependent between about pH 7.5 and pH 11.9 (see above). The pH dependence of azurin is not described by a simple 1H^+^/1e^−^ Nernstian model, indicating that multiple sites are involved with the pH dependence [24]. This is corroborated by the work on charge regulation in azurin and in other proteins [33,34]. Overall, the change in the p*K*_a_ of surface residues was shown to correlate with the total reorganization energy (likely outer-sphere) in several metalloproteins. With respect to the RuHis83-azurin studied here, it seems that any of the changes in driving force and reorganization energy are small in order to produce proportionally small changes in observed and calculated ET rate constants.

The lack of pH dependence on Cu^+^ oxidation lower than pH 8 for Ru-His83-azurin is worth brief comments. Ullmann and co-workers have explored, in detail, the underpinnings of the pH-dependent reduction of Cu^+^ in azurin, using experimental data as a benchmark [32]. In particular, there exists a high probability of His83 and His35 being protonated upon reduction at pH values between 6 and 8. In Ru-His83-azurin, the binding of the Ru-label at His83 blocks that site from protonation, but His35 remains accessible. In Ullman’s work, they demonstrate that protonation of His35 upon Cu^+^ reduction is coupled to a peptide flip. In this context, one could expect more pH dependence; however, we lack more detailed mechanistic information. The lack of pH dependence in Ru-His83-azurin might suggest that proton transfer or peptide flip occurs after Cu^2+^ oxdidation, but more work is clearly needed to make firm conclusions.

**Figure 4:**
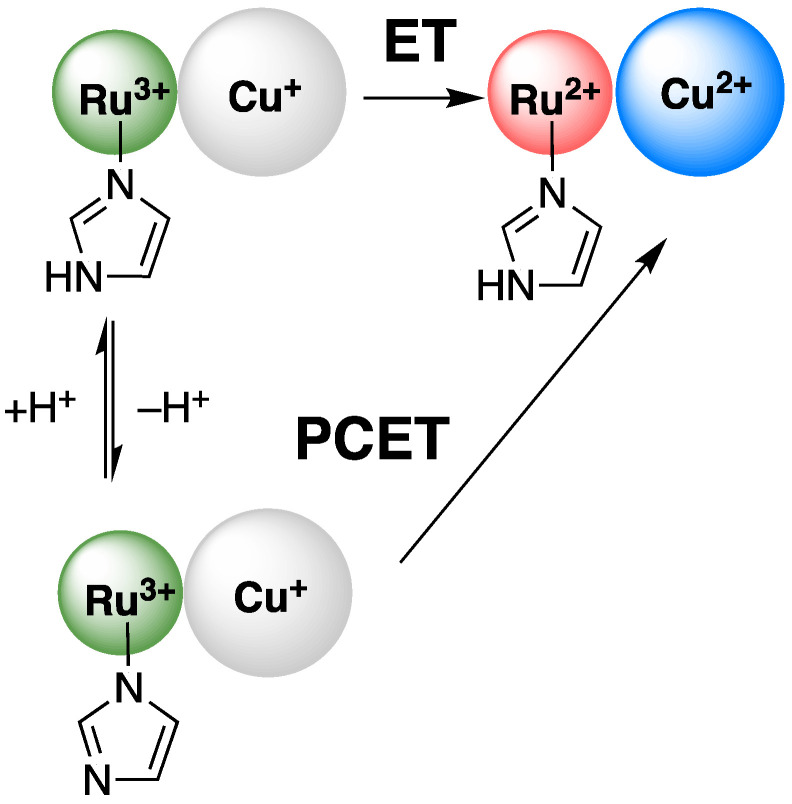
Schematic of the pure-electron transfer (ET) and the proton-coupled electron transfer (PCET) pathways for the oxidation of Cu^+^ by Ru^3+^-His83 oxidants.

As noted above, the discrepancy between experiment and theory is more pronounced at pH 9 and pH 9.5. At these pH values, the reduction potentials for azurin are pH-independent, so it must be the redox behavior of the Ru label that contributes to the observed change in *k*_ET_. There are two limiting possibilities. Both possibilities involve the oxidation of Cu^+^ by a deprotonated Ru^3+^, that is, an imidazolate- or histidinate-ligated Ru^3+^ (Figure 4). The p*K*_a_ values of oxidized complexes are always lower (more acidic) than the corresponding reduced complexes [53,54]. Thus, it can be expected that, following the flash quench reaction, the Ru^3+^ label will rapidly deprotonate. The first possibility is that Cu^+^ is oxidized by Ru^3+^-imidazolate to form Cu^2+^ and a Ru^2+^ imidazolate which is subsequently re-protonated. The value for *E*° (Ru^3+^-imidazolate/Ru^2+^-imidazolate) can be estimated as 0.47 V versus SHE using our data and literature p*K*_a_ values [43]. The calculated value of *k*_ET_ is between 5.2 × 10^5^ and 1.0 × 10^6^ s^−1^ for values of λ between 0.7 and 0.8 eV. The calculated rate constants are in good agreement with experimental work.

The second option is that the reduction of Ru^3+^-imidazolate occurs via concerted addition of H^+^ and e^−^ in one step. This has been called, equivalently, “concerted proton-electron transfer” or “electron proton transfer” [54,55]. Such reactions are known to have larger associated values of λ [56,57]. Thus, it is straightforward to use Equation (1) to calculate pH-dependent λ values with ET rate constants and driving forces fixed at our experimental values. These values are set out in Table 2. The increase in λ is reasonable based on other literature examples [56,57], especially one involving ET and PCET of a ruthenium diimine complex [57].

To the best of our knowledge, there is only one other example of the pH dependence of intramolecular ET in azurin [58]. In broad terms, those authors observe the same general trend, with larger ET rate constants at low pH values. However, the magnitude of the change in *k*_ET_ is larger (over a factor of 10). We emphasize that those authors use a different system in which the kinetics of Cu^2+^ reduction by disulfide radicals are monitored. The authors discuss the pH dependence of the Cu^2+/+^ couple in azurin, but any pH dependence of disulfide redox reactions is not considered explicitly. As is the case for our Ru-modified system, the rate constants cannot be satisfactorily modeled using Equation (1) with fixed values of H_AB_ and/or λ. Ultimately, the authors propose small changes to the degree of electronic coupling (H_AB_).

Taking the observations from reference [58] in account, as well as our results from Ru-His83 azurin, we propose that the pathways shown Figure 4 are the likely origin of the changes in ET rate constant as a function of pH in both modified and unmodified azurins. A change in mechanism to PCET with larger associated values of λ, or significant changes in reduction potentials due to deprotonation, give rise to similar decreases in *k*_ET_ as pH is increased. With respect to λ, and as is expected from the Marcus additivity postulate, if the reorganization energy of one redox site changes, the value of λ for the entire system changes and thus the observed rate constant.

The results from this study underscore the importance of PCET in biological redox systems. The concept of charge regulation [33,34] provides a means to rationalize pH-dependent reduction potentials and outer-sphere reorganization energy. By spreading the changes to surface p*K*_a_ across several weakly coupled sites, biomolecules can have a less pronounced (non-Nernstian) pH dependence. This also maintains the naturally low reorganization energy of biological metal sites. In the RU-modified azurin studied here, the pH dependence of the Ru site has a strong pH dependence and therefore a larger impact of ET rate constants. At near-neutral pH values, any change in mechanism is compensated by an increase in driving force (Table 1), but at high pH Cu^+^, oxidation likely occurs via a different mechanism, which is reflected in the observed rate constants. Ultimately, it seems that biomolecules have evolved coupled pathways that control both electron and proton transfers, even in “simple” electron carriers like azurin.

## 4. Materials and Methods

All chemicals were from Sigma-Aldrich and used as received, unless otherwise noted. Ru(2,2′-bipyridyl)_2_Cl_2_ was from Strem Chemicals. The complexes Co(2,2′-bipyridyl)_3_(PF_6_)_2_ and Ru(2,2′-bipyridyl)_2_CO_3_ were prepared according to the literature [59,60]. Ru(NH_3_)_6_Cl_3_ was recrystallized before use [61]. All buffer salts were from Fisher Scientific and used as received. BL21-DE3 *E. coli* were from Thermo-Fisher Scientific. All media for purification of protein samples was from GE Healthcare (Cytiva). Protein samples were concentrated using an Amicon ultrafiltration cell under N_2_ positive pressure with Sartorius Hydrosart 10K ultrafiltration membranes. All experiments utilized purified water purified to a resistivity of 18 MΩ cm^−1^ using a Barnstead EASYpure system. The APB buffer used in all variable pH experiments comprised 20 mM each of sodium acetate, dibasic potassium phosphate, and sodium borate, as well as 100 mM potassium chloride. The pH of each solution was adjusted using 1 M HCl or 1 M KOH, as appropriate.

Optical spectra were collected using a Cary 100 Bio UV-Visible spectrophotometer. Electrochemical experiments were carried out using a standard three-electrode setup with an edge plane graphite working electrode, a glassy carbon counter electrode, and a silver/silver chloride reference electrode. Cobalt(III) tris(2,2′-pipyridyl) was used as an internal standard. Cyclic voltammetry data were collected using a CH Instruments CHI6171B Electrochemical Analyzer.

The plasmid for expression of wild-type *Pseudomonas aeruginosa* azurin was a gift from Harry B. Gray and John H. Richards (California Institute of Technology). Azurin was expressed and purified according to the literature. modification with Ru(bpy)_2_ at His83 was carried out as previously described. Final protein purification used a Pharmacia fast protein liquid chromatography system. Azurin concentrations were assessed using UV-vis spectroscopy and the known molar absorptivity of the azurin charge transfer band ε (628 nm) = 5600 M^−1^ cm^−1^.

The model complex Ru(bpy)_2_(im)_2_(PF_6_) was synthesized following a literature procedure (bpy = 2,2′-bipyridiyl). To facilitate dissolution of the PF_6_^−^ salts for electrochemistry in water, concentrated DMSO stocks were prepared and diluted to a final concentration of 0.1 mM in APB buffers at each pH value. Azurin samples also were assayed at 0.1 mM concentrations. Cyclic voltammograms were collected using scan rates of 100 mV s^−1^. The working electrode was polished between each scan with 0.3 µm alumina micropolish powder and a microfiber lab (Buheler). For measurements of azurin, potassium ferricyanide (K_3_Fe(CN)_6_) was employed as an external standard and measurements of Ru(bpy)_2_(im)_2_(PF_6_)_2_ used Co(bpy)_3_(PF_6_) as an internal standard.

Transient absorption experiments were conducted using a home-built spectrometer that has been described elsewhere. The excitation of the samples was achieved using a 480 nm laser beam with an energy of 6 ± 1 mJ per pulse. Protein samples were reduced with ascorbic acid, desalted into the appropriate pH buffer and deoxygenated with a 15–20 pump-backfill cycles with N_2_ gas. Initial luminescence decay traces were collected prior to adding Ru(NH_3_)_6_Cl_3_ to a final concentration of 12–18 mM; the concentration did not affect the observed ET rate constants. Samples were again deoxygenated following the addition of quencher and time-resolved luminescence, and absorbance data were collected. The resulting kinetics traces were analyzed using the MATLAB (version R2023b) Curve Fitting toolbox.

## Figures and Tables

**Table 1:** Summary of experimental driving forces and ET rate constants and the corresponding calculated ET rate constants.

pH	−∆G° ^1^	*k*_ET_ (×10^−6^) ^2^	*k*_ET,calc_ (×10^−6^) ^3^
4	−0.671	1.2 ± 0.1	2.18
5	−0.671	1.4 ± 0.1	2.18
6	−0.673	1.3 ± 0.1	2.18
7	−0.698	1.3 ± 0.1	2.20
8	−0.693	1.3 ± 0.1	2.20
9	−0.655	0.3 ± 0.1	2.14
9.5	−0.634	0.4 ± 0.1	2.07

^1^ Errors in −∆G° values are ±0.01 V. ^2^ Calculated values of *k*_ET_ use Equation (1) and fixed values of λ (0.7 eV), the Ru-Cu distance (17 Å), H_AB_ at close contact is 186 cm^−1^, and the distance decay constant (β = 1.1 Å^−1^). ^3^ Errors in *k*_ET,calc_ are approximately ±2 × 10^4^ s^−1^ based on errors in −∆G°.

**Table 2:** Calculated reorganization energy (λ).

pH	λ ^1^
4	0.88
5	0.85
6	0.87
7	0.89
8	0.85
9	1.10
9.5	1.03

^1^ Values are computed using Equation (1) with rate constants and driving forces fixed to the experimental values in Table 1.

## Data Availability

Data from this study are uploaded as Supporting Information.

## Supplementary Materials

The following supporting information can be downloaded at: https://www.mdpi.com/article/10.3390/molecules30030472/s1, Figure S1. Representative cyclic voltammograms of [Ru(bpy)_2_(im)_2_](PF_6_)_2_ (0.1 mM) in 20 mM APB buffer at the pH values indicated. The wave at 0.3 V is for 0.1 mM [Co(bpy)_3_]^3+/2+^ internal standard. The pH values are as indicated in each panel. Figure S2. Representative cyclic voltammograms of wild-type azurin (0.1 mM) in 20 mM APB buffer at the pH values indicated. The external standard was 0.1 mM [Co(bpy)_3_]^3+/2+^. The pH values are as indicated in each panel. Table S1. Ru^2+/3+^ reduction potentials for [Ru(2,2´-bipyridyl)_2_(imidazole)_2_](PF_6_)_2_. The values are versus SHE and are taken from the cyclic voltammetry data shown in Figure S1. The values for azurin are taken from the literature (see main text) and confirmed with selected data shown in Figure S2. Figure S3. UV-Visible spectrum of Ru-His83 azurin. The band at 628 nm is the Cys-Cu(II) charge transfer transition in azurin. The bands at 490 and 350 nm are charge transfer transitions from the Ru(bpy)_2_(imidazole)his83) label. The concentration of the sample is 35 µM. Figure S4. Illustration of the sequence of photochemical and redox reactions involved in flash-quench generation of Ru^III^-His83 oxidants. In this study, Q = Ru(NH_3_)_6_^3+^, though in principle many different electron acceptors can be used. The step labeled as “ET” is the reaction of interest. Kinetics traces for this reaction are shown below. Figure S5. Representative time-resolved kinetics traces (628 nm) of Ru-His83 azurins. The concentrations for each trace were 30 µM protein in 20 mM APB buffer at the pH values indicated. The concentration of the quencher, Ru(NH_3_)_6_Cl_3_ was 12 mM. The apparent bleach at early times arises from luminescence from unquenched (residual) *Ru^II^. The pH values are as indicated in each panel. Table S2. Summary of experimental driving forces and ET rate constants and the corresponding calculated ET rate constants.

## References

[1] Minnihan E.C., Nocera D.G., Stubbe J. (2013). Reversible, Long-Range Radical Transfer in *E. coli* Class Ia Ribonucleotide Reductase. Acc. Chem. Res..

[2] Winkler J.R., Gray H.B. (2014). Electron Flow through Metalloproteins. Chem. Rev..

[3] Wikström M., Krab K., Sharma V. (2018). Oxygen Activation and Energy Conservation by Cytochrome c Oxidase. Chem. Rev..

[4] Beratan D.N. (2019). Why Are DNA and Protein Electron Transfer So Different?. Annu. Rev. Phys. Chem..

[5] Blumberger J. (2018). Electron Transfer and Transport through Multi-Heme Proteins: Recent Progress and Future Directions. Curr. Opin. Chem. Biol..

[6] Tommos C. (2022). Insights into the Thermodynamics and Kinetics of Amino-Acid Radicals in Proteins. Annu. Rev. Biophys..

[7] Cukier R.I., Nocera D.G. (1998). Proton-Coupled Electron Transfer. Annu. Rev. Phys. Chem..

[8] Dempsey J.L., Winkler J.R., Gray H.B. (2010). Proton-Coupled Electron Flow in Protein Redox Machines. Chem. Rev..

[9] Stubbe J., Nocera D.G., Yee C.S., Chang M.C.Y. (2003). Radical Initiation in the Class I Ribonucleotide Reductase: Long-Range Proton-Coupled Electron Transfer?. Chem. Rev..

[10] Mayer J.M. (2004). Proton-Coupled Electron Transfer: A Reaction Chemist’s View. Annu. Rev. Phys. Chem..

[11] Warren J.J., Mayer J.M. (2015). Moving Protons and Electrons in Biomimetic Systems. Biochemistry.

[12] Dekker J., Van G. (2000). Primary Charge Separation in Photosystem II. Photosynth. Res..

[13] Kern J., Alonso-Mori R., Tran R., Hattne J., Gildea R.J., Echols N., Gl√∂ckner C., Hellmich J., Laksmono H., Sierra R.G. (2013). Simultaneous Femtosecond X-Ray Spectroscopy and Diffraction of Photosystem II at Room Temperature. Science.

[14] Umena Y., Kawakami K., Shen J.-R., Kamiya N. (2011). Crystal Structure of Oxygen-Evolving Photosystem II at a Resolution of 1.9Å. Nature.

[15] Warren J.J., Herrera N., Hill M.G., Winkler J.R., Gray H.B. (2013). Electron Flow through Nitrotyrosinate in Pseudomonas Aeruginosa Azurin. J. Am. Chem. Soc..

[16] Warren J.J., Shafaat O.S., Winkler J.R., Gray H.B. (2016). Proton-Coupled Electron Hopping in Ru-Modified P. Aeruginosa Azurin. J. Biol. Inorg. Chem..

[17] Cordes M., Köttgen A., Jasper C., Jacques O., Boudebous H., Giese B. (2008). Influence of Amino Acid Side Chains on Long-Distance Electron Transfer in Peptides: Electron Hopping via “Stepping Stones”. Angew. Chem. Int. Ed..

[18] Cordes M., Giese B. (2009). Electron Transfer in Peptides and Proteins. Chem. Soc. Rev..

[19] Hay S., Westerlund K., Tommos C. (2005). Moving a Phenol Hydroxyl Group from the Surface to the Interior of a Protein: Effects on the Phenol Potential and pKA. Biochemistry.

[20] Martínez-Rivera M.C., Berry B.W., Valentine K.G., Westerlund K., Hay S., Tommos C. (2011). Electrochemical and Structural Properties of a Protein System Designed To Generate Tyrosine Pourbaix Diagrams. J. Am. Chem. Soc..

[21] Nilsen-Moe A., Reinhardt C.R., Huang P., Agarwala H., Lopes R., Lasagna M., Glover S., Hammes-Schiffer S., Tommos C., Hammarström L. (2024). Switching the Proton-Coupled Electron Transfer Mechanism for Non-Canonical Tyrosine Residues in a de Novo Protein. Chem. Sci..

[22] Fedoretz-Maxwell B.P., Shin C.H., MacNeil G.A., Worrall L.J., Park R., Strynadka N.C.J., Walsby C.J., Warren J.J. (2022). The Impact of Second Coordination Sphere Methionine-Aromatic Interactions in Copper Proteins. Inorg. Chem..

[23] Berry S.M., Baker M.H., Reardon N.J. (2010). Reduction Potential Variations in Azurin through Secondary Coordination Sphere Phenylalanine Incorporations. J. Inorg. Biochem..

[24] Clair C.S.S., Ellis Jr W.R., Gray H.B. (1992). Spectroelectrochemistry of Blue Copper Proteins: pH and Temperature Dependences of the Reduction Potentials of Five Azurins. Inorg. Chim. Acta.

[25] Magliozzo R.S., McIntosh B.A., Sweeney W.V. (1982). Origin of the pH Dependence of the Midpoint Reduction Potential in Clostridium Pasteurianum Ferredoxin: Oxidation State-Dependent Hydrogen Ion Association. J. Biol. Chem..

[26] Zu Y., Fee J.A., Hirst J. (2001). Complete Thermodynamic Characterization of Reduction and Protonation of the Bc1-Type Rieske [2Fe-2S] Center of *Thermus thermophilus*. J. Am. Chem. Soc..

[27] Reid L.S., Taniguchi V.T., Gray H.B., Mauk A.G. (1982). Oxidation-Reduction Equilibrium of Cytochrome B5. J. Am. Chem. Soc..

[28] Reid L.S., Mauk M.R., Mauk A.G. (1984). Role of Heme Propionate Groups in Cytochrome B5 Electron Transfer. J. Am. Chem. Soc..

[29] Pettigrew G.W., Meyer T.E., Bartsch R.G., Kamen M.D. (1976). pH Dependence of the Oxidation-Reduction Potential of Cytochrome *c*_2_. Biochim. Biophys. Acta—Bioenerg..

[30] Moore G.R., Pettigrew G.W., Pitt R.C., Williams R.J.P. (1980). pH Dependence of the Redox Potential of *Pseudomonas Aeruginosa* Cytochrome *c*-551. Biochim. Biophys. Acta—Bioenerg..

[31] Battistuzzi G., Borsari M., Sola M. (2001). Redox Properties of *Cytochrome* c. Antioxid. Redox Signal..

[32] Ullmann R.T., Ullmann G.M. (2011). Coupling of Protonation, Reduction, and Conformational Change in Azurin from Pseudomonas Aeruginosa Investigated with Free Energy Measures of Cooperativity. J. Phys. Chem. B.

[33] Zahler C.T., Zhou H., Abdolvahabi A., Holden R.L., Rasouli S., Tao P., Shaw B.F. (2018). Direct Measurement of Charge Regulation in Metalloprotein Electron Transfer. Angew. Chem. Int. Ed..

[34] Zahler C.T., Shaw B.F. (2019). What Are We Missing by Not Measuring the Net Charge of Proteins?. Chem. Eur. J..

[35] Zhang A.Y., Koone J.C., Dashnaw C.M., Zahler C.T., Shaw B.F. (2020). Complete Charge Regulation by a Redox Enzyme Upon Single Electron Transfer. Angew. Chem. Int. Ed..

[36] Lund M., Jönsson B. (2005). On the Charge Regulation of Proteins. Biochemistry.

[37] Ullmann G.M. (2000). The Coupling of Protonation and Reduction in Proteins with Multiple Redox Centers:  Theory, Computational Method, and Application to Cytochrome C3. J. Phys. Chem. B.

[38] Ullmann G.M., Bombarda E. (2013). pKa Values and Redox Potentials of Proteins. What Do They Mean?. Biol. Chem..

[39] Bombarda E., Ullmann G.M. (2010). pH-Dependent pKa Values in Proteins—A Theoretical Analysis of Protonation Energies with Practical Consequences for Enzymatic Reactions. J. Phys. Chem. B.

[40] Warburton R.E., Soudackov A.V., Hammes-Schiffer S. (2022). Theoretical Modeling of Electrochemical Proton-Coupled Electron Transfer. Chem. Rev..

[41] Faham S., Day M.W., Connick W.B., Crane B.R., Di Bilio A.J., Schaefer W.P., Rees D.C., Gray H.B. (1999). Structures of Ruthenium-Modified Pseudomonas Aeruginosa Azurin and [Ru(2,2′-Bipyridine)2(Imidazole)2]SO_4_·10H_2_O. Acta Crystallogr. D Biol. Crystallogr..

[42] Krishnan C.V., Brunschwig B.S., Creutz C., Sutin N. (1985). Homogeneous Catalysis of the Photoreduction of Water. 6. Mediation by Polypyridine Complexes of Ruthenium(II) and Cobalt(II) in Alkaline Media. J. Am. Chem. Soc..

[43] Long C., Vos J.G. (1984). Acid-Base Chemistry of Some 1,2,4-Triazole and Imidazole Complexes of Ruthenium(II)Bis(2,2′-Bipyridyl). Inorganica Chim. Acta.

[44] Reddy K.B., Cho M.P., Wishart J.F., Emge T.J., Isied S.S. (1996). *cis*-Bis(Bipyridine)Ruthenium Imidazole Derivatives: A Spectroscopic, Kinetic, and Structural Study. Inorg. Chem..

[45] Yuan Z., Yang H., Malik N., Čolović M., Weber D.S., Wilson D., Bénard F., Martin R.E., Warren J.J., Schaffer P. (2019). Electrostatic Effects Accelerate Decatungstate-Catalyzed C–H Fluorination Using [^18^F]- and [^19^F]NFSI in Small Molecules and Peptide Mimics. ACS Catal..

[46] Gibbs C.A., Ghazi N., Tao J., Warren J.J. (2024). An Investigation of the Influence of Tyrosine Local Interactions on Electron Hopping in a Model Protein. Molecules.

[47] Skov L.K., Pascher T., Winkler J.R., Gray H.B. (1998). Rates of Intramolecular Electron Transfer in Ru(Bpy)2(Im)(His83)-Modified Azurin Increase below 220 K. J. Am. Chem. Soc..

[48] Gray H.B., Winkler J.R. (2003). Electron Tunneling through Proteins. Q. Rev. Biophys..

[49] Malmström B.G., Wittung-Stafshede P. (1999). Effects of Protein Folding on Metalloprotein Redox-Active Sites: Electron-Transfer Properties of Blue and Purple Copper Proteins. Coord. Chem. Rev..

[50] Winkler J.R., Wittung-Stafshede P., Leckner J., Malmström B.G., Gray H.B. (1997). Effects of Folding on Metalloprotein Active Sites. Proc. Natl. Acad. Sci. USA.

[51] Di Bilio A.J., Hill M.G., Bonander N., Karlsson B.G., Villahermosa R.M., Malmstroem B.G., Winkler J.R., Gray H.B. (1997). Reorganization Energy of Blue Copper: Effects of Temperature and Driving Force on the Rates of Electron Transfer in Ruthenium- and Osmium-Modified Azurins. J. Am. Chem. Soc..

[52] Gray H.B., Winkler J.R. (2005). Long-Range Electron Transfer. Proc. Natl. Acad. Sci. USA.

[53] Agarwal R.G., Coste S.C., Groff B.D., Heuer A.M., Noh H., Parada G.A., Wise C.F., Nichols E.M., Warren J.J., Mayer J.M. (2022). Free Energies of Proton-Coupled Electron Transfer Reagents and Their Applications. Chem. Rev..

[54] Warren J.J., Tronic T.A., Mayer J.M. (2010). Thermochemistry of Proton-Coupled Electron Transfer Reagents and Its Implications. Chem. Rev..

[55] Weinberg D.R., Gagliardi C.J., Hull J.F., Murphy C.F., Kent C.A., Westlake B.C., Paul A., Ess D.H., McCafferty D.G., Meyer T.J. (2012). Proton-Coupled Electron Transfer. Chem. Rev..

[56] Roth J.P., Lovell S., Mayer J.M. (2000). Intrinsic Barriers for Electron and Hydrogen Atom Transfer Reactions of Biomimetic Iron Complexes. J. Am. Chem. Soc..

[57] Kessinger M., Soudackov A.V., Schneider J., Bangle R.E., Hammes-Schiffer S., Meyer G.J. (2022). Reorganization Energies for Interfacial Proton-Coupled Electron Transfer to a Water Oxidation Catalyst. J. Am. Chem. Soc..

[58] Farver O., Bonander N., Skov L.K., Pecht I. (1996). The pH Dependence of Intramolecular Electron Transfer in Azurins. Inorganica Chim. Acta.

[59] Hamann T.W., Gstrein F., Brunschwig B.S., Lewis N.S. (2005). Measurement of the Dependence of Interfacial Charge-Transfer Rate Constants on the Reorganization Energy of Redox Species at n-ZnO/H_2_O Interfaces. J. Am. Chem. Soc..

[60] Johnson E.C., Sullivan B.P., Salmon D.J., Adeyemi S.A., Meyer T.J. (1978). Synthesis and Properties of the Chloro-Bridged Dimer [(Bpy)_2_RuCl]_2_^2+^ and Its Transient 3+ Mixed-Valence Ion. Inorg. Chem..

[61] Meyer T.J., Taube H. (1968). Electron-Transfer Reactions of Ruthenium Ammines. Inorg. Chem..

