# Supplementary material for: The Influence of pH on Long-Range Electron Transfer and Proton-Coupled Electron Transfer in Ruthenium-Modified Azurin
Source: Molecules. 2025 Jan 22;30(3):472. doi: 10.3390/molecules30030472 (PMC11821252; doi:10.3390/molecules30030472)
Supplement: Supplementary file 1 [file molecules-30-00472-s001.zip › molecules-3429071-supplementary.pdf]

Supporting Information for:

The Influence of pH on Long-Range Electron Transfer and Proton-Coupled Electron Transfer in Ruthenium-Modified Azurin

Nikta Ghazi and Jeffrey J. Warren

Department of Chemistry
Simon Fraser University
8888 University Drive
Burnaby BC, Canada, V5A 1S6
*jjwarren@sfu.ca

Contents

1. Cyclic voltammograms of [ruthenium(II))2,2´-bipyridyl)$_2$(imidazole)$_2$](PF$_6$)$_2$ 2
2. Cyclic voltammograms of azurin at selected pH values 4
3. Summary of reduction potentials for [ruthenium(II))2,2´-bipyridyl)$_2$(imidazole)$_2$](PF$_6$)$_2$ 5
4. Example flash-quench kinetics scheme 7
5. Representative transient absorbance traces for RuHis83-azurin 8
6. Calculated ET rate constants with an alternate value of λ 10

1. Cyclic voltammograms of [ruthenium(II))2,2′-bipyridyl)$_2$(imidazole)$_2$](PF$_6$)$_2$

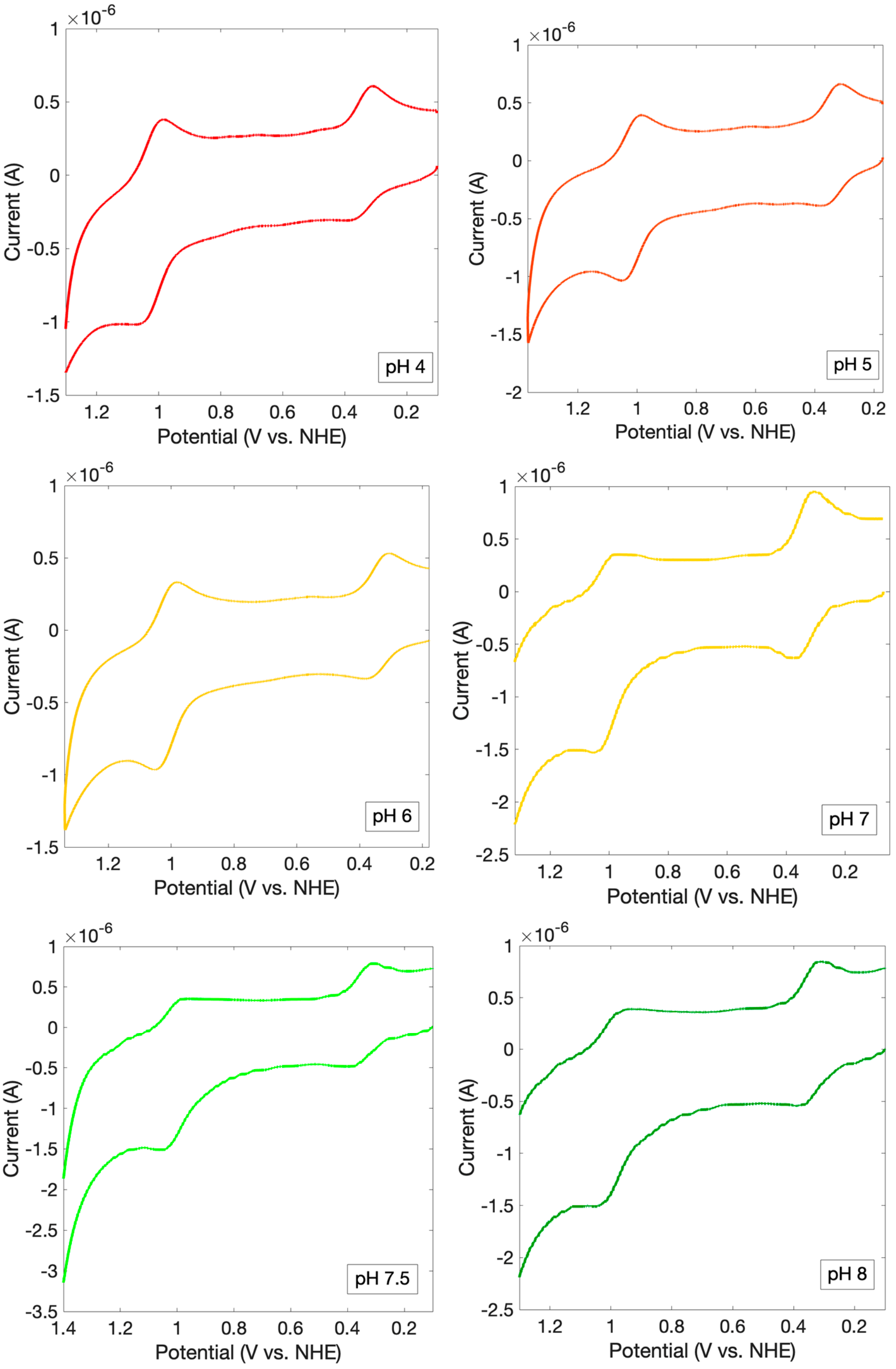

Figure S1 (continues below). Representative cyclic voltammograms of [Ru(bpy)$_2$(im)$_2$](PF$_6$)$_2$ (0.1 mM) in 20 mM APB buffer at the pH values indicated. The wave at 0.3 V is for 0.1 mM [Co(bpy)$_3$]$^{3+/2+}$ internal standard. The pH values are as indicated in each panel.

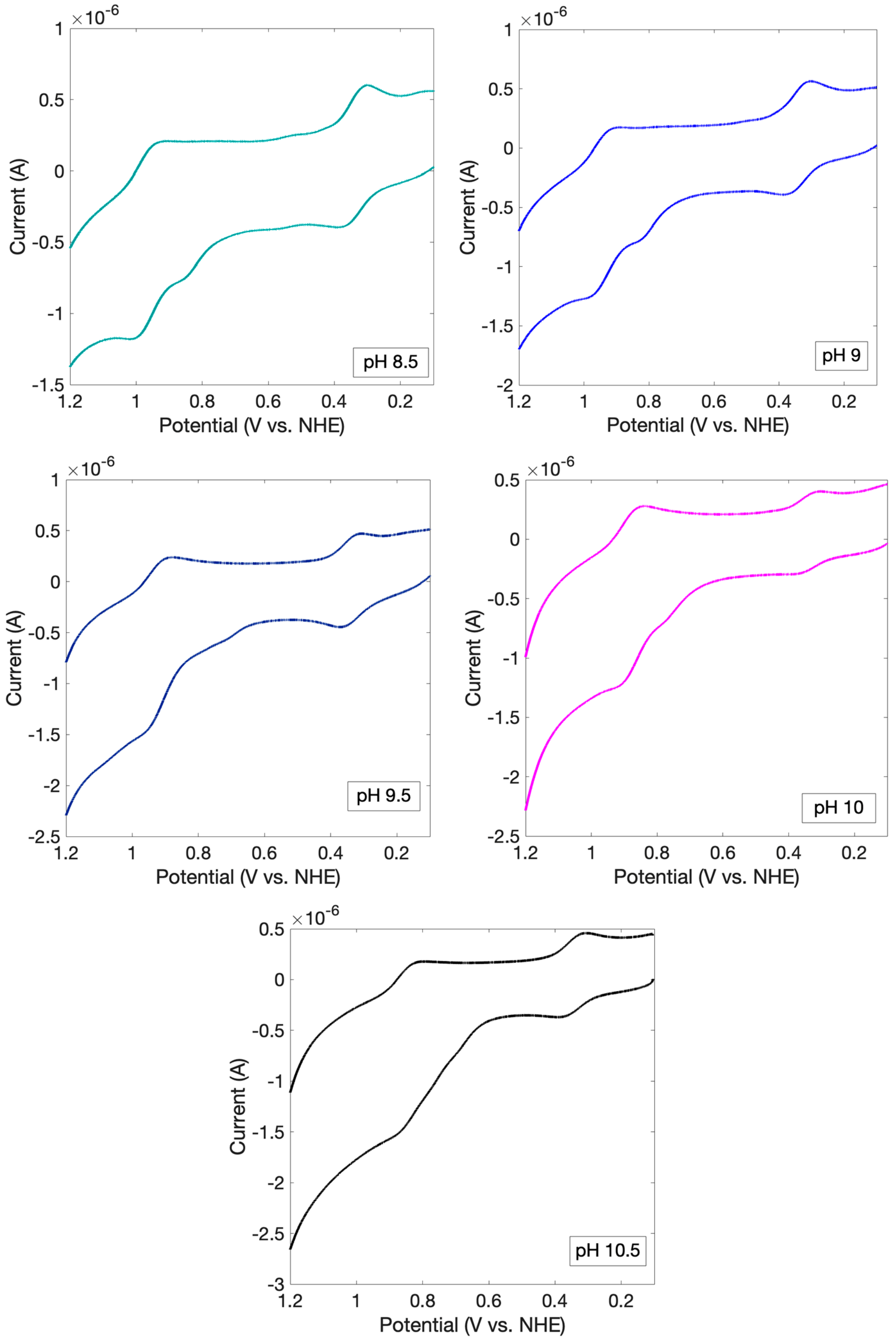

Figure S1 (continues from above). Representative cyclic voltammograms of $[Ru(bpy)_2(im)_2](PF_6)_2$ (0.1 mM) in 20 mM APB buffer at the pH values indicated. The wave at 0.3 V is for 0.1 mM $[Co(bpy)_3]^{3+/2+}$ internal standard. The pH values are as indicated in each panel.

2. Cyclic voltammograms of azurin at selected pH values

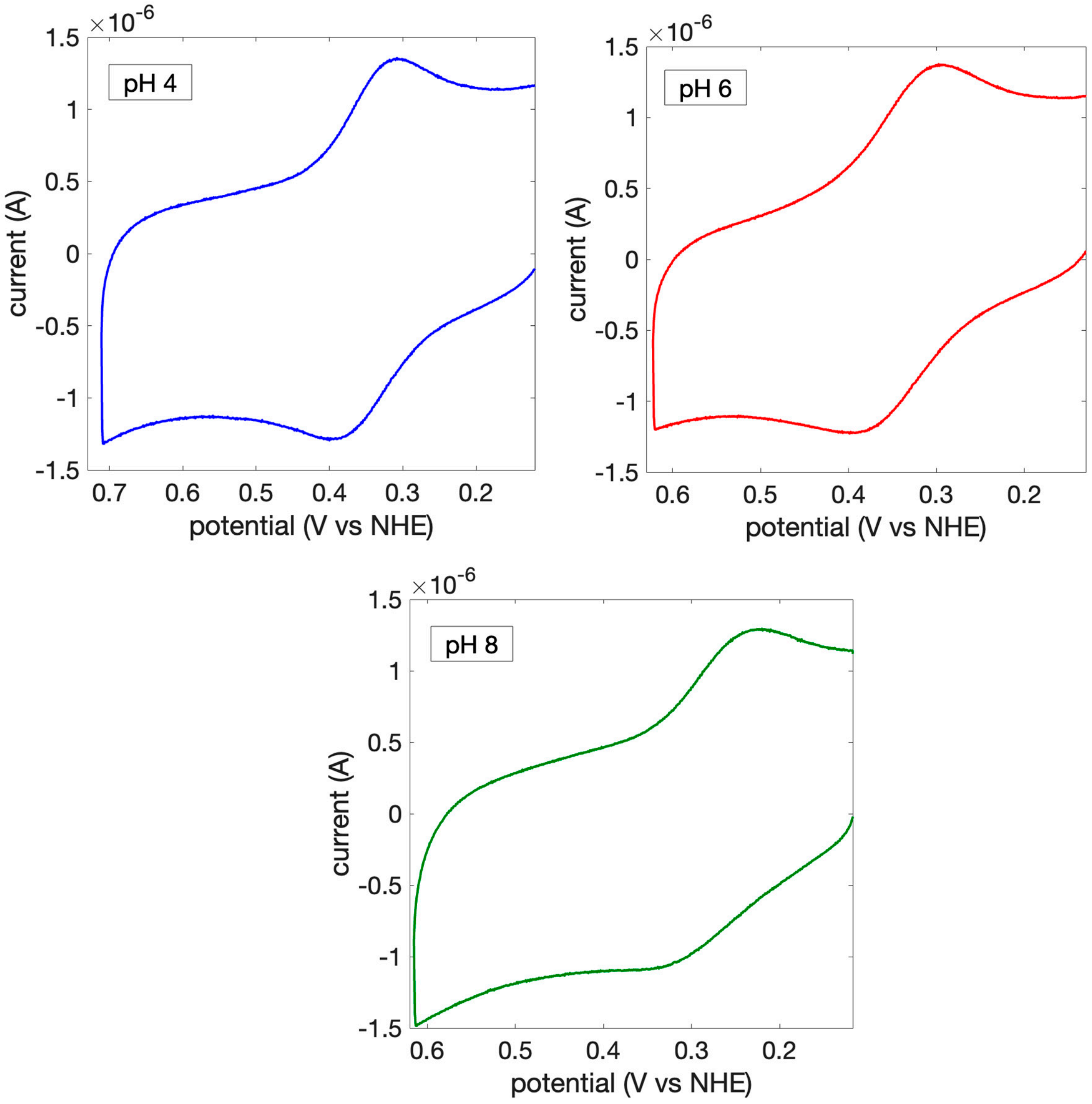

Figure S2. Representative cyclic voltammograms of wild-type azurin (0.1 mM) in 20 mM APB buffer at the pH values indicated. The external standard was 0.1 mM $[Co(bpy)_3]^{3+/2+}$. The pH values are as indicated in each panel.

3. Summary of reduction potentials for [ruthenium(II))2,2´-bipyridyl)$_2$(imidazole)$_2$](PF$_6$)$_2$ and for azurin

Table S1. $Ru^{2+/3+}$ reduction potentials for [Ru(2,2´-bipyridyl)$_2$(imidazole)$_2$](PF$_6$)$_2$. The values are versus SHE and are taken from the cyclic voltammetry data shown in Figure S1. The values for azurin are taken from the literature (see main text) and confirmed with selected data shown in Figure S2.

pH	$E_{1/2}(Ru^{III/II})$	$E_{1/2}$(Azurin-$Cu^{2+/+}$)
4	1.020	0.348
5	1.019	0.347
6	1.012	0.339
7	1.010	0.311
7.5	1.009	0.299
8	0.985	0.292
8.5	0.969	0.289
9	0.944	0.288
9.5	0.922	0.288
10	0.875	N/D
10.5	0.849	N/D

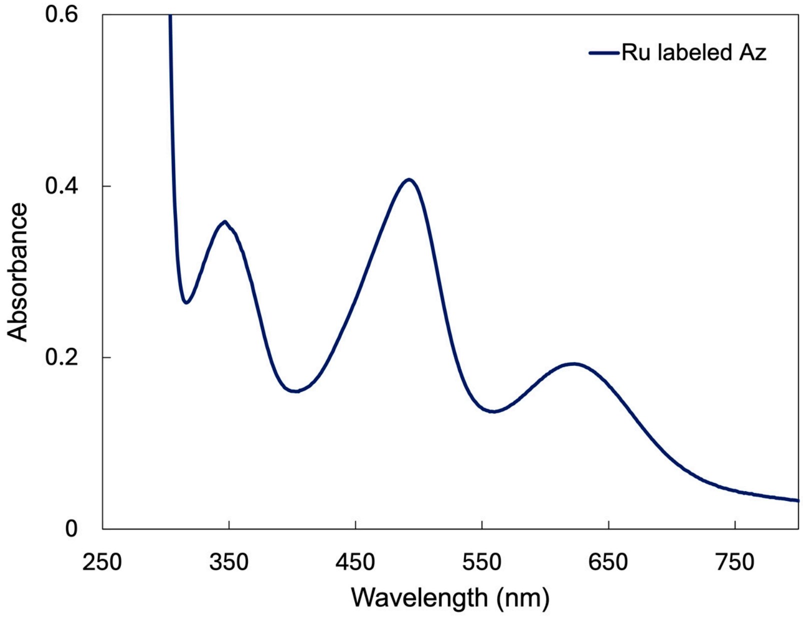

Figure S3. UV-Visible spectrum of Ru-His83 azurin. The band at 628 nm is the Cys-Cu(II) charge transfer transition in azurin. The bands at 490 and 350 nm are charge transfer transitions from the Ru(bpy)$_2$(imidazole)his83) label. The concentration of the sample is 35 μM.

4. Example flash-quench kinetics scheme

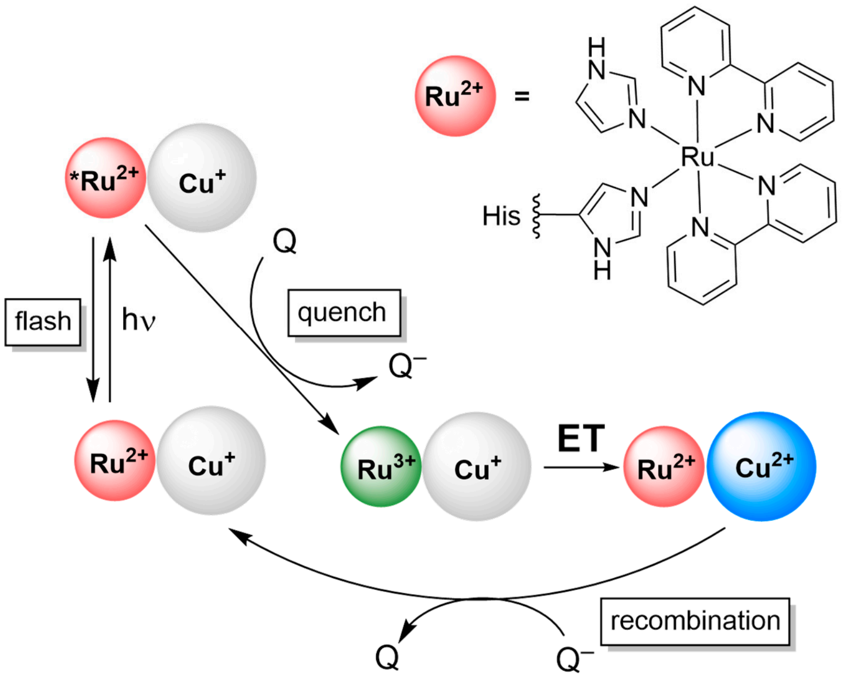

Figure S4. Illustration of the sequence of photochemical and redox reactions involved in flash-quench generation of Ru^{III}-His83 oxidants. In this study, Q = $Ru(NH_3)_6^{3+}$, though in principle many different electron acceptors can be used. The step labeled as "ET" is the reaction of interest. Kinetics traces for this reaction are shown below.

5. Representative transient absorbance traces for RuHis83-azurin

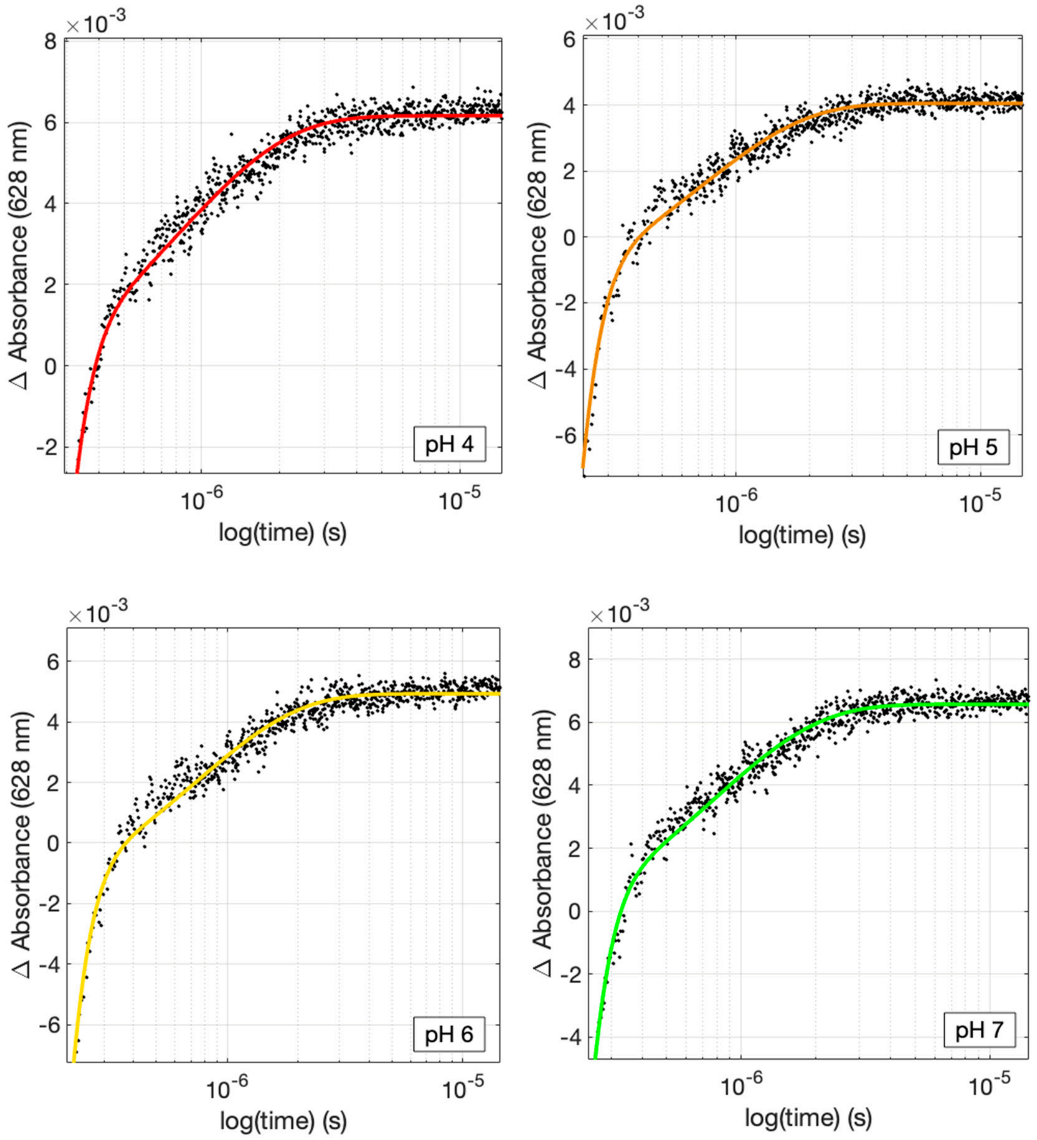

Figure S5 (continues below). Representative time-resolved kinetics traces (628 nm) of Ru-His83 azurins. The concentrations for each trace were 30 μM protein in 20 mM APB buffer at the pH values indicated. The concentration of the quencher, $Ru(NH_3)_6Cl_3$ was 12 mM. The apparent bleach at early times arises from luminescence from unquenched (residual) $*Ru^{II}$. The pH values are as indicated in each panel.

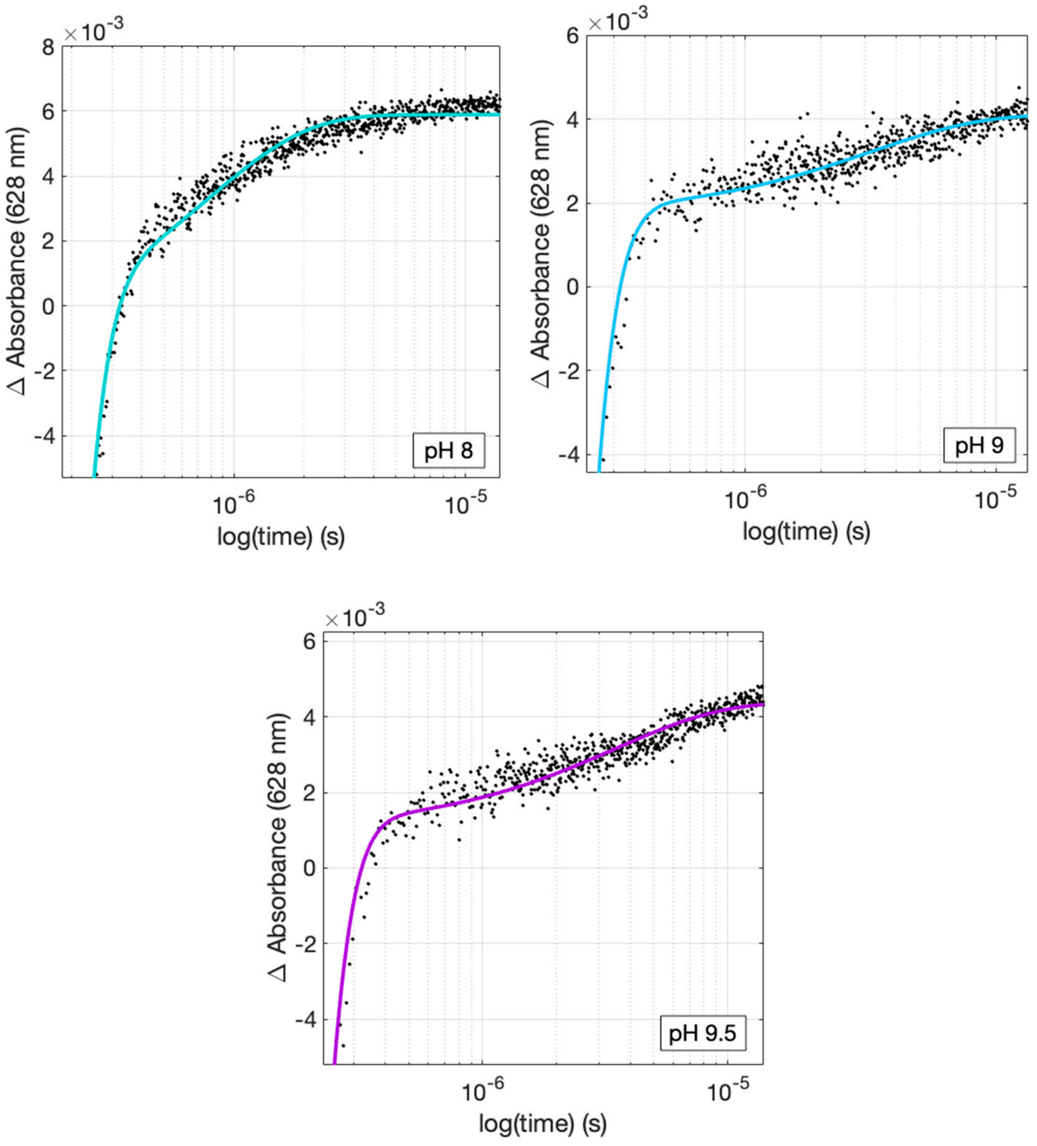

Figure S5 (continues from above). Representative time-resolved kinetics traces (628 nm) of Ru-His83 azurins. The concentrations for each trace were 30 μM protein in 20 mM APB buffer at the pH values indicated. The concentration of the quencher, $Ru(NH_3)_6Cl_3$ was 12 mM. The apparent bleach at early times arises from luminescence from unquenched (residual) $^*Ru^{II}$. The pH values are as indicated in each panel.

6. Calculated ET rate constants with an alternate value of λ

Table S2. Summary of experimental driving forces and ET rate constants and the corresponding calculated ET rate constants

	$-\Delta G°$	k_{ET} ($\times 10^{-6}$)	$k_{ET, calc}$ ($\times 10^{-6}$)
4	–0.671	1.2 ± 0.1	1.68
5	–0.671	1.4 ± 0.1	1.68
6	–0.673	1.3 ± 0.1	1.69
7	–0.698	1.3 ± 0.1	1.81
8	–0.693	1.3 ± 0.1	1.79
9	–0.655	0.3 ± 0.1	1.59
9.5	–0.634	0.4 ± 0.1	1.47

[1] Calculated values of k_{ET} use equation 1 and fixed values of λ (0.8 eV), the Ru-Cu distance (17 Å), H_{AB} at close contact is 186 cm^{-1}, and the distance decay constant (β = 1.1 $Å^{-1}$).
